# Improving quality of reproductive health care in Senegal through formative supervision: results from four districts

**DOI:** 10.1186/1478-4491-5-26

**Published:** 2007-11-29

**Authors:** Siri Suh, Philippe Moreira, Moussa Ly

**Affiliations:** 1 University of Michigan Population Fellow, Management Sciences for Health Cambridge, MA 02139, USA; 2 Management Sciences for Health, Cambridge, MA 02139, USA

## Abstract

**Background:**

In Senegal, traditional supervision often focuses more on collection of service statistics than on evaluation of service quality. This approach yields limited information on quality of care and does little to improve providers' competence. In response to this challenge, Management Sciences for Health (MSH) has implemented a program of formative supervision. This multifaceted, problem-solving approach collects data on quality of care, improves technical competence, and engages the community in improving reproductive health care.

**Methods:**

This study evaluated changes in service quality and community involvement after two rounds of supervision in 45 health facilities in four districts of Senegal. We used checklists to assess quality in four areas of service delivery: infrastructure, staff and services management, record-keeping, and technical competence. We also measured community involvement in improving service quality using the completion rates of action plans.

**Results:**

The most notable improvement across regions was in infection prevention.

Management of staff, services, and logistics also consistently improved across the four districts. Record-keeping skills showed variable but lower improvement by region. The completion rates of action plans suggest that communities are engaged in improving service quality in all four districts.

**Conclusion:**

Formative supervision can improve the quality of reproductive health services, especially in areas where there is on-site skill building and refresher training. This approach can also mobilize communities to participate in improving service quality.

## Background

In 1994, the International Conference on Population and Development set in motion a global movement to promote the reproductive health and rights of women, men and young people. Although significant progress has been made, many challenges to improving reproductive health outcomes remain in the developing world [1]. Unmet need for modern contraception in estimated at 29% in developing countries. Nearly all (99%) of the 529 000 maternal deaths occurring each year around the world take place in developing countries. Although most maternal deaths are related to unexpected complications, only half of all births worldwide are attended by health workers with the skills required to provide emergency obstetric care. Each year, nearly 5 million people are newly infected with HIV. Nearly half of all adults living with HIV/AIDS are women and in sub-Saharan Africa, almost 60% of HIV-positive adults are women [1]. In recent years, a growing recognition of the importance of quality of care in improving sexual and reproductive health has emerged. Although evidence shows that access to services is crucial to improving reproductive health outcomes, health programmers and policymakers are increasingly aware that successful reproductive health strategies must also address service quality [1-3].

Part of the response to the imperative of improved quality of care has been the emergence of alternative forms of supervision. In contrast to traditional models that have a limited focus on data collection and analysis of results, these new approaches focus on joint problem-solving, immediate feedback, and communication between supervisor and provider. Management Sciences for Health (MSH) is implementing formative supervision, an innovative approach to supervision of reproductive health care that involves the community, in Senegal. This paper describes the formative supervision approach and evaluates changes in service quality after two rounds of formative supervision in four districts of Senegal.

### Supervision and quality

Approaches to improving quality of care have usually focused on training providers and upgrading infrastructure and equipment [4], and better supervision of providers has often been a part of these strategies [5]. In her framework for quality of care of reproductive health, Bruce points to supervision as an underpinning of technical competence [6]. Supervision entails a range of activities, including observation of providers' performance, data collection, and a reinforcement of job descriptions, skills, institutional norms and protocols. Beyond these technical elements, in some settings supervision plays an important role in personalizing the health system for service providers, whose contact with the administration of the health system is often limited to supervisory visits [5]. Taken together, these activities compose the four main objectives of supervision as defined by US Agency for International Development's (USAID's) Maximizing Access and Quality Initiative: setting expectations, monitoring and evaluation of performance, identifying problems and opportunities for improvement, and mobilizing action [5].

In many settings, supervision takes the limited form of inspecting performance against checklists. This fault-finding approach may demoralize health workers and undermine joint problem-solving and action, so health programmers and providers favor forms of supervision that focus on addressing problems in service delivery [5,7,8]. One such approach, developed by EngenderHealth and called "facilitative supervision", emphasizes a comprehensive analysis of the factors that shape a provider's ability to perform his or her job. This approach emphasizes mentoring, joint problem-solving, and open communication [9].

### Evidence for alternative approaches to supervision

Evidence from program evaluations and research studies in various countries suggests that facilitative or supportive supervision promotes service quality. In six countries–Bangladesh, Brazil, Honduras, Kenya, Nepal, and Tanzania–the introduction of supportive supervision as part of service improvement initiatives has yielded promising results in both service quality and provider performance [5]. Research findings offer more rigorous support for alternative forms of supervision. One of the earliest examples is taken from the 1980s in Brazil, where adoption of a self-assessment approach to supervision at a community-based family planning distribution program not only improved performance, but also increased the number of providers supervised and reduced the cost of supervision [10]. Studies in Guatemala, Mexico, and Indonesia have also noted the effectiveness of self-assessment as a supervisory tool [11-13]. Other studies in Zimbabwe, Nigeria, Nepal, and Malawi indicate that structured observation using checklists and immediate feedback also leads to improved performance [14-17].

### The situation in Senegal

In Senegal, there is a significant need for high-quality sexual and reproductive health care. The contraceptive prevalence rate for modern methods is low (10%) [18]. Although 93% of women receive prenatal care, skilled providers attend just over half of all births (52%) [18].

Norms and protocols for reproductive health care, defined by the Ministry of Health, state the objectives, tools, and frequency required for supervision. According to these standards, the objectives of supervision are to provide refresher training, improve working conditions, and motivate and support health workers. Supervisors are supposed to use checklists to assess working conditions and the technical competence of staff. Supervisors are also expected to evaluate the job descriptions of various categories of providers [19]. Community health structures are supervised every month, while health posts are supervised every two months. Facilities at the district and regional levels receive supervision every three and six months, respectively. However, evidence suggests serious lapses in the observation of supervision protocols. The frequency of supervision by local authorities is often inconsistent, and the tools and activities associated with supervision are not applied in a standard fashion at all health facilities due to resource and organizational constraints [20,21]. One study found that in the last six months, most facilities (60%) received one or two visits. However, nearly 31% of facilities did not receive any supervision. Under these conditions, the capacity of supervision to improve service quality has been limited.

In addition, under the classic supervision system, the data available is often difficult to analyse. For example, family planning and maternal health data is often expressed in terms of the availability, accessibility, and utilization of services by region [22]. While an interregional comparison of the data is interesting, it provides a limited understanding of quality of care and the technical competence of providers in each region. It is also unclear what these indicators represent and how they are measured. Availability may represent the availability of services, or the number of health workers trained to provide services. Accessibility can refer to financial or geographic access, or even cultural acceptability of services. The data fails to provide the information needed to develop activities geared toward improving quality of care.

### The formative supervision intervention in Senegal

To address the gap between information and programming to improve quality and to reinforce the technical competence of providers, we implemented formative supervision. This type of supportive supervision combines observation with a problem-solving approach to clinical, logistic, and information, education, and communication (IEC) problems in health service delivery. This approach differs from other supportive supervision approaches in two ways. Firstly, formative supervision draws on a range of tools and activities designed to assess the technical competence of providers in the delivery of reproductive health care. Secondly, formative supervision includes the community in the supervision process by orienting community representatives towards a rights-based approach to service quality.

Using the Ministry's Norms and Protocols for Sexual and Reproductive Health of 2000, in 2002 we developed a checklist to evaluate the quality of sexual and reproductive health care. Partners included the United Nations Population Fund, the United Nations Fund for Children, the World Health Organization, and USAID and several of its cooperating agencies. Local nongovernmental organizations such as Santé Familiale (Family Health, or SANFAM) and l'Association Sénégalaise pour le Bien-Etre de la Famille (Senegalese Association for the Well-Being of the Family, or ASBEF) also participated in developing the checklist.

We pre-tested the checklist at several health facilities in the regions of Louga and Thiès in 2003. Since then, we have implemented formative supervision in various districts of USAID's six intervention regions. In each district, all health posts and health centers offering reproductive health services were selected to receive a total of two supervision visits over the course of the program. A total of 323 facilities in six regions were visited during the first round of supervision. The second round of supervision began in July 2005 in the region of Thiès. Table 1 shows where formative supervision was implemented in the six intervention regions between 2003 and 2005.

**Table 1:** Formative supervision in six regions of Senegal, 2003–2005

	**Visit 1, 2003–2005**						**Visit 2, 2005**					
**Region**	**Districts**	**Health centers**	**Health posts**	**Reference centers**	**Maternities**	**Total**	**Districts**	**Health centers**	**Health posts**	**Reference centers**	**Maternities**	**Total**

Dakar	3	0	10	0	2	12	0	0	0	0	0	0
Fatick	1	1	4	0	0	5	0	0	0	0	0	0
Kaolack	4	4	52	0	0	56	0	0	0	0	0	0
Louga	5	5	61	1	0	67	2	1	21	0	0	22
Thiès	7	8	98	0	0	106	2	3	20	0	0	23
Ziguinchor	4	2	73	0	2	77	0	0	0	0	0	0

**Total**	**24**	**20**	**298**	**1**	**4**	**323**	**4**	**4**	**41**	**0**	0	**45**

### Formative supervision tools

Formative supervision uses four tools to assess quality of care with a problem-solving approach: the supervision checklist, the infection prevention exercise, the COPE exercise [23], and the Inventory Management Assessment Tool (IMAT) [24]. The checklist and IMAT obtain quantitative data on provider and facility performance. The infection prevention and COPE exercises, both qualitative tools, mobilize providers and community members to collaborate to evaluate service quality and identify solutions for quality improvement.

As in other supervisory approaches, much of the supervision visit revolves around the completion of the checklist. Supervisors use direct observation to compare performance to the checklist and provide immediate feedback to providers. The checklist used for formative supervision is different from other checklists in three respects. First, it integrates the clinical, logistic, and IEC components of service quality by evaluating the following indicators:

• Availability and quality of infrastructure and equipment;

Human resources and services management (organization and availability of health services; record-keeping; community involvement; functionality of the facility's management committee; and functionality of the community-based health committee);

• Technical competence of health workers in providing clinical and IEC services;

• Accuracy in drug supply record-keeping and effectiveness of stock management.

**Figure 1:**
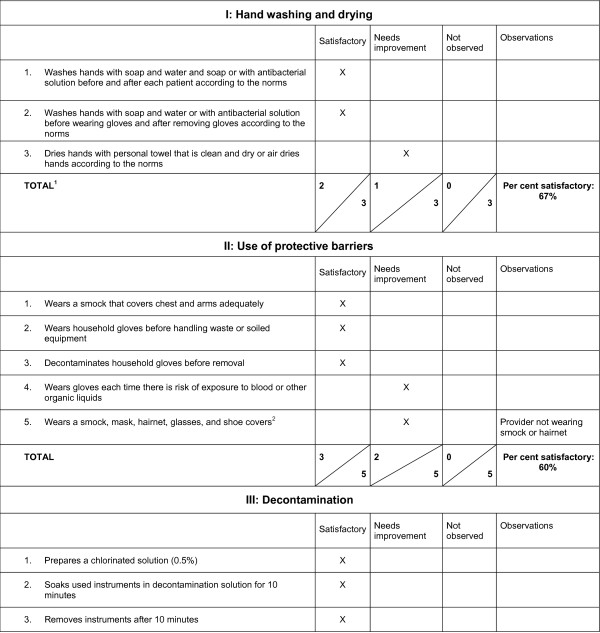
**Example of page 1 of infection prevention in supervision checklist**. 1. The number of checked boxes in each column determines the total score for each section, e.g., in the section on hand washing and drying, 2 items were marked "Satisfactory" and 1 item was marked "Needs improvement." The total score is 2/3 or 67%. We determine the total score for Infection Prevention by adding all the items marked "Satisfactory" and dividing by the total number of items in the section. 2. If all the conditions in a multiple component question are not observed, the supervisor marks the "Needs improvement" column and makes remarks in the "Observations" column to provide necessary feedback.

Second, by including sections appropriate for supervision of various types of facilities, the checklist is adaptable to the range of health facilities in the national health care system: health posts, health centers, regional hospitals, and national hospitals. For example, the checklist of essential drugs is more extensive for hospitals than for health posts. Third, the checklist calculates quantifiable measures of performance for immediate feedback. For each area of reproductive health service delivery, we calculate a score according to the points gained out of the total number of points on the checklist during the observation period. We then convert the score to a percentage that can be analysed at the facility, district, and regional levels. Figure 1 illustrates the completed first page of the section on infection prevention in the checklist.

The second tool used during formative supervision is a live demonstration of the infection prevention exercise adapted from the EngenderHealth model. Supervisors demonstrate four steps of infection prevention: handwashing; use of protective barriers (gloves); treatment of instruments (decontamination, cleaning, sterilization, and high-level disinfection); and elimination of waste.

Using buckets, gloves, and various cleaning agents, the supervisor explains the concept and importance of infection prevention to providers and community members. Providers are invited to demonstrate their infection prevention skills to the audience. The supervisor identifies opportunities for improvement in the providers' techniques and encourages questions and feedback from the audience. Community members are encouraged to participate not only to observe, but also to mobilize community support for the purchase of infection prevention supplies.

Next, the COPE exercise orients clients and providers to a rights-based approach to reproductive health service delivery. Using materials adapted from the EngenderHealth model, providers complete self-assessments to evaluate their own performance. Supervisors administer questionnaires to clients to assess their perceptions of service delivery. Drawing on the data collected from these tools, the supervisors lead a group discussion with providers and community representatives on rights-based concepts of service delivery from the perspectives of both clients and providers. The supervisors then combine the highlights of this discussion with their observations from the checklist to guide the development of action plans for community members and providers to improve quality of care.

**Figure 2:**
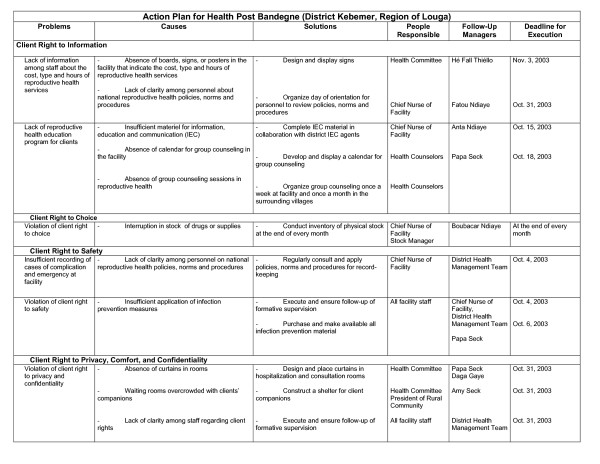
Action plan for health post Bandegne (District Kebemer, Region of Louga).

Figure 2 illustrates the first page of an action plan completed in 2003 at a health facility in the district of Kebemer in Louga. Action plans include tasks that fall under the COPE model, including the right of the client to information, choice, safety, privacy, comfort, and confidentiality. Plans also address the human resource needs of health providers. The action plan committee designates who is responsible for completing each task. While providers are responsible for improving technical areas of service delivery such as stock management, infection prevention, and clinical management, the community often shares the responsibility for improving or upgrading the facility and its equipment. Nearly every facility has a health committee, which is responsible for representing community interests. Health committees also often assume responsibility for mobilizing financial support or labor from the community. Communities have repaired or constructed incinerators for elimination of medical waste, constructed signs with prices and hours of service, and erected road signs indicating the location and services of the nearest health facility.

The participation of community members in the process of quality improvement is perhaps the most innovative aspect of formative supervision. To assess community involvement, we used the completion rates of action plans developed collaboratively by providers and community members during supervision visits. Completion rates serve as an indirect measure of the participation of community members in helping to complete tasks related to quality improvement.

The fourth tool used in formative supervision is the Inventory Management Assessment Tool (IMAT). Developed by MSH in Haiti in 1997, the IMAT is used to assess the accuracy of stock registration and the effectiveness of drug supply management for up to 25 commonly used drugs. Table 2 lists the IMAT indicators. By examining both stock records and physical stock, supervisors obtain the data required to calculate the IMAT indicators. Stock managers are invited to participate so that they learn to use the tool themselves, and supervisors share the results with them to identify strategies for improving inventory record-keeping and management. In addition to applying IMAT, supervisors often assist stock managers in physically reorganizing storage units to facilitate identification and storage of medical supplies.

**Table 2:** Integrated management assessment tool indicators

**Indicator**	**Definition**	**Desired level**
**Accuracy of stock registration system**		

1. Percentage of accurate stock registration	Indicates the quality of the stock registration system	100%
1a. Percentage of recorded stock less than physical stock	Indicates proportion of recorded stock balance less than physical stock balance	0%
1b. Percentage of recorded stock greater than physical stock	Indicates proportion of recorded stock balance greater than physical stock balance	0%
2. Ratio of inventory variation to total stock (expressed in percentages)	Indicates the extent of registration errors	0%

**Effectiveness of stock maintenance system**		

3. Percentage of products in stock	Measures the system's capacity to maintain a complete range of products in stock at the time of the assessment	100%
4. Average percentage of time that products are out of stock	Indicates the system's capacity to maintain a constant supply of products over time by minimizing the duration of stock-outs	0%

## Methods

We used two primary sources of data to assess how formative supervision affected service quality and community involvement in improving service quality. To measure changes in service quality between the two rounds of supervision, we calculated percentages of satisfactory performance in the areas defined in the supervision checklist. To measure community involvement in improving service quality, we analysed the completion of action plans developed collaboratively by health providers and community members.

### Assessing service quality

#### Health districts included in the study

We collected and analyzed data from the application of the checklists in two rounds of supervision in the districts of Tivaoune and Khombole in the region of Thiès and the districts of Kebemer and Louga in the region of Louga. Of the more than 300 health facilities in these four districts, 45 facilities received two supervision visits: 23 in Tivaoune and Khombole and 22 in Kebemer and Louga. The total population covered by the district of Tivaoune is 185 250; in Khombole, the population covered is 244 000. The district of Kebemer covers a population of 149 444; in Louga, the population covered is 340 472.

#### Areas of service delivery included in the study

We specified four areas of service delivery in the analysis: infrastructure, management of staff and services, record-keeping, and technical competence. The checklist contains indicators of quality for each area of service delivery. *Infrastructure *refers to the condition of the facility and its surrounding, the state of equipment and supplies, and the physical layout of the facility. *Management of staff and services *refers to human resource management strategies and tools, such as the existence of job descriptions and event calendars, appropriate delegation of tasks, and integration of health services. *Record-keeping *represents the maintenance of registers and patient records for family planning, prenatal care, and delivery care. *Technical competence *measures providers' performance in family planning and prenatal care consultations, individual and group counseling, infection prevention, and logistics management.

#### Selection of facilities

We selected 45 facilities in the four districts of Tivaoune, Khombole, Louga and Kebemer for the analysis. The selection of these facilities was not random. Rather, these facilities were included in the analysis because they had received two supervision visits. In addition, the number of facilities differs for each area of service delivery because we included only facilities where performance in that particular area was observed during both rounds of supervision. Table 3 displays the health facilities included in the analysis for each area of service delivery according to type of facility and region. For example, the analysis of technical competence in infection prevention includes only those facilities where infection prevention skills were observed during both supervision visits. Facilities where infection prevention was observed during the first visit or the second visit only were not included. Technical competence in logistics management is the only area of service delivery where performance was observed in all 45 facilities during both two supervision visits.

**Table 3:** Health facilities included in analysis by area of service delivery and by type

	**Region of Thiès**			**Region of Louga**			
**Area of service delivery**	**Health posts**	**Health centers**	**Total**	**Health posts**	**Health centers**	**Total**	**Total**

**Infrastructure**	19	3	22	21	1	22	44
**Organization of services**	18	3	21	19	1	20	41
**Record-keeping**							
**Family planning tools:**							
Patient files	17	3	20	9	1	10	30
Registers	19	3	22	14	1	15	37
Maternity tools:							
Prenatal care register	20	3	23	9	1	10	33
Delivery register	17	3	20	10	1	11	31

**Technical competence**:							
Prenatal care	13	3	16	10	0	10	26
Family planning	2	1	3	5	0	5	8
Individual counselling	4	1	5	4	0	4	9
Group counselling	2	1	3	0	0	0	3
Infection prevention	12	2	14	16	0	16	30
Logistics management	20	3	23	21	1	22	45

#### Checklist analysis

Using the number of satisfactory responses from the checklist, we calculated percentages of performance for each facility in the four areas of service delivery from both rounds of supervision. Table 4 displays the average performance of all four districts during both rounds of supervision. For each district, the table indicates the number of facilities included in the analysis of the four areas of service delivery. We derived district performance in each service from the combined average of all facilities in the district that were observed during both rounds of supervision. Percentages for performance for the first and second supervision visits are listed in Table 4 under the columns labeled '2003' and '2005,' respectively. The differences in average performance between the first and second rounds of supervision are shown for each district.

**Table 4:** Health facility performance in four areas of service delivery

	**Region of Thiès**									**Region of Louga**								
	**Tivaoune District performance (%)**				**Khombole District performance (%)**				**Change in regional performance (%)**	**Kebemer District performance (%)**				**Louga District performance (%)**				**Change in regional performance (%)**

	**Facilities**	**2003**	**2005**	**Difference**	**Facilities**	**2003**	**2005**	**Difference**		**Facilities**	**2003**	**2005**	**Difference**	**Facilities**	**2003**	**2005**	**Difference**	

**Infrastructure**	12	54	58	4	10	54	58	5	4	11	60	65	5	11	56	59	3	4
																		
**Services/staff management**	12	34	68	34	9	26	39	13	23	10	53	70	17	10	46	60	15	16
																		

**Record-keeping**																		

**Family planning tools**																		
Patient files	12	90	88	-2	8	68	85	17	7	6	69	87	19	4	67	92	25	22
Registers	13	76	80	4	9	61	76	15	9	8	56	76	20	7	57	69	12	16
																		
**Maternity tools**																		
Prenatal care register	13	93	93	0	10	78	89	11	5	5	75	80	4	5	87	77	-10	-3
Delivery room register	11	71	70	-1	9	63	74	12	5	4	64	77	13	7	65	76	11	12

																		

**Technical competence**																		

Prenatal care consultation	11	55	49	-6	5	55	51	-4	-5	4	44	64	20	6	45	57	13	16
Family planning consultation	3	30	40	10	0	/	/	/	Unavailable*	1	33	49	16	4	32	48	16	16
Individual counselling	5	49	67	17	0	/	/	/	Unavailable	1	43	66	23	3	50	64	14	19
Group counselling	2	41	74	33	1	46	65	20	26	0	/	/	/	0	/	/	/	Unavailable
Infection prevention	8	34	60	26	6	15	46	31	28	7	20	53	33	9	18	49	30	32
Logistics management	13	45	64	19	10	22	62	40	29	11	57	65	8	11	50	62	12	10

* We are unable to report a percentage of change for regions where there were districts with no facilities in which performance was observed during both supervision visits. This is the case in district of Khombole for technical competence in family planning consultation and individual counselling and in Kebemer and Louga for technical competence in group counselling.

Table 4 also displays the regional changes in performance calculated by averaging district measures of change. In the district of Khombole in the areas of family planning consultation and individual counseling, there were no facilities in which performance for these two indicators was observed during both visits. We are therefore unable to report average district performance and the difference in performance between rounds of supervision. Since data for one district is unavailable, we cannot report the regional change in performance for these two indicators. The same is true for the districts of Kebemer and Louga in the area of group counseling. We did not perform tests to determine statistical significance because our sample of facilities was not randomly selected.

#### Assessing community participation

During the first round of supervision carried out in 2003–2005, providers and community representatives developed action plans to improve the quality of service. In 2005, we initiated follow-up visits to assess progress in the execution of the action plans. We analyzed action plans in the four districts to evaluate community participation in service improvement. In the region of Thiès, we included 14 action plans from Tivaoune and 9 from Khombole in the analysis. In the region of Louga, we included 9 action plans from Kebemer and 15 from Louga. We calculated completion rates for each facility by dividing the number of tasks completed by the total number of tasks planned. For example, the action plan included in Figure 2 had a completion rate of 12 out of 18 tasks, or 67%. The 6 tasks that were not fully completed at the time of follow-up visits were the design of signs indicating cost, type, and hours of services; the development of a monthly schedule for group counseling and the execution of group counseling sessions at the facility; the purchase of all required infection prevention material; the purchase of all material required for functional facility beds; and the construction of a shelter for clients' companions.

We calculated district rates of completion by averaging the rates of individual health facilities in each district. We obtained regional rates of completion by averaging district measures.

## Results

### Service quality

Table 4 displays the results from the analysis of checklist data. Overall, the data suggests improvement in the four quality of care indicators across regions and districts. The most remarkable change across regions was in technical competence in infection prevention, with Thiès improving by 28% and Louga by 32%. This is a critical finding given that performance in infection prevention was among the lowest in all areas of technical competence during the first round of supervision. While data on technical competence in group counseling was unavailable for the region of Louga, data showed providers in the region of Thiès improved by 26%. In the region of Thiès, performance in logistics management improved by 29%. In the region of Louga, technical competence in prenatal care consultation improved by 16%, although performance declined in quality in both districts in the region of Thiès. Family planning was another area of technical competence where strikingly low levels of performance were observed during the first supervision visit. Skills in family planning consultation improved in the region of Louga by 16%, and in the district of Tivaoune by 10%.

Progress was observed in both regions in management of staff and services, with Thiès improving by 23% and Louga improving by 16%. The smallest change observed in both regions was in infrastructure, with both Thiès and Louga improving by 4%. During both rounds of supervision, facilities in all four districts consistently performed better in record-keeping for family planning and maternity services than in any other area of service delivery. During the first round, all facilities scored above 56%. Although minor reductions in record-keeping skills were observed in Tivaoune and Louga during the second round, performance in record-keeping in all four districts generally remained well above performance in other areas of service delivery during both rounds of supervision.

### Community involvement in improving quality

Table 5 illustrates results from the analysis of action plans. The data suggest that community members are engaged in activities designed to improve service quality. Completion rates of action plans ranged from 33% in the district of Khombole to 67% in the district of Louga. The average regional execution rate for Louga (62%) was higher than for Thiès (48%).

**Table 5:** Completion of action plans after first round of formative supervision

	**Region of Thiès**			**Region of Louga**		
	**Tivaoune**	**Khombole**	**Regional Average**	**Louga**	**Kebemer**	**Regional Average**

Number of action plans included in analysis	14	9	12	15	9	12
Average rate of completion (%)	63	33	48	56	67	62

## Discussion

In resource-poor settings, where supervision often revolves around the collection of data from facility registers and patient records without addressing the challenges involved in service delivery, formative supervision offers a useful approach to improving reproductive health care. With the flexibility to draw on various tools and activities, formative supervision facilitates a comprehensive assessment of the quality of reproductive health care. Formative supervision focuses on technical competence and provides a forum for addressing areas in need of improvement. Where a classic supervision approach may provide limited data on quality of care and virtually none on the technical competence of providers, formative supervision has yielded critical data on specific areas of service provision.

The findings of this study with those of other studies suggest that supportive supervision can improve service quality. We observed improvements in infrastructure, management of staff and services, record-keeping and technical competence. The most notable improvements–in the areas of infection prevention, logistics management, and counseling–may be linked to the unique set of problem-solving tools applied during formative supervision.

Numerous factors could account for the variations in improvements between districts in areas of service provision such as infrastructure, record-keeping, and technical competence. Although MSH provides logistical support to health facilities in the form of donated equipment and supplies, facilities are responsible for the cost of purchasing needed equipment. Communities also contribute by purchasing supplies or by providing resources for upgrading facilities. The modest improvements in infrastructure observed in all four districts may reflect the limited financial capacity of health structures or resource mobilization constraints that exist at community level. Many action plans have identified insufficient financial resources as a barrier to quality service delivery. The minor reduction in record-keeping performance observed in two districts may correspond to the difficulty in obtaining improvements when competence is already high.

The decline in prenatal competence that occurred in Thiès may be explained by a deficiency in the prenatal care section of the checklist used during the second round of supervision in Thiès. In Senegal, national norms and protocols require providers to give pregnant women two doses of sulfadoxine pyrimethamine (three pills per dose) to prevent malaria during the second and third trimesters of pregnancy [25]. Known as intermittent preventive treatment, these doses must be taken in the presence of a health provider. The checklist used in Thiès did not sufficiently define the management of intermittent preventive treatment during the appropriate trimesters of pregnancy. Providers' performance in Thiès may thus have been underestimated during the second supervision visit. This problem was rectified after the second round of supervision in Thiès, and a checklist that correctly defined intermittent preventive treatment for malaria was administered in the region of Louga. The new checklist has been used for subsequent supervision visits in intervention zones.

One of the most promising aspects of formative supervision is the participation of the community in evaluating and improving service quality. Through their involvement in developing action plans, community representatives are able to voice their concerns and contribute financially to improving their health facilities. In this study, completion rates of action plans served as an indirect measure of community involvement in health care. While this measure is interesting, efforts should be directed to finding more direct means of assessing community participation. Conducting qualitative research with community members responsible for executing action plans would be one approach. Developing a system to track the mobilization of community resources for service improvement would be another. Supervisors have already noted that more local health committees are purchasing bleach in response to the infection prevention exercise. At some facilities, in response to the lack of protection against malaria identified by supervisors, health committees have purchased window netting or insecticide-impregnated mosquito nets for maternity wards to protect women and newborns. Future measures of community involvement must take into account financial and other resources invested in service improvement.

This study was subject to some limitations. The small sample size is attributable to the inclusion of only facilities that received both rounds of supervision in the analysis. The limited sample size is most noticeable in the area of technical competence, where only facilities in which providers' performance was observed during both visits were included in the analysis (Table 3). The ability to observe consultations and other demonstrations of technical competence is related to fluctuations in client flow. Some facilities were excluded from the analysis because no consultations were observed during supervision visits. The frequency of supervision is another serious limitation. Although supervision visits are supposed to occur every six months, in reality the second round of supervision for the regions of Thiès and Louga did not occur until nearly three years after the first. Much of this delay can be attributed to the challenges involved in organizing two-day supervision visits with health authorities in the 24 districts included in MSH's intervention zone. Increasing the frequency of supervision would raise the likelihood of observing technical consultations during a visit, thereby increasing the number of facilities eligible for evaluation. Changes in human resources in both regions between supervision visits constitute another limitation of this study. Due to unforeseen absences and transfers of staff determined by district health authorities, in some facilities a different provider was evaluated during each supervision visit. The evaluation of different providers may have invalidated some results between rounds of supervision.

The personal biases of supervisors constituted another limitation of this study. Although evaluation of service quality is based on national standards and protocols, varying perceptions of quality among supervisors may have biased the completion of the checklist. Changes in supervisors between rounds of supervision may have also contributed to bias due to differences in personal perceptions. Other limitations stem from the design of our study. Although formative supervision permits a comprehensive evaluation of various aspects of quality of reproductive health care, our analysis focused on the evaluation of certain indicators in health facilities. Thus, we are unable to detect an overall improvement of service quality in reproductive health care at the facility level. In addition, the non-random selection of health facilities for the analysis precludes the generalization of findings to other facilities.

In spite of these limitations, we are using the lessons learned from this experience to improve the formative supervision process. Efforts are being made to reduce the amount of turnover in supervisors during visits. To increase the frequency of visits, we have adopted a more targeted approach to formative supervision that reduces the duration of each visit from two days to one. Supervisors study checklists and IMAT data from previous visits to identify areas in need of improvement at each facility. During the second visit, supervisors focus evaluation activities on those problem areas. The checklist has also been streamlined to make data collection faster. In addition, supervisors exercise discretion in applying the COPE and infection prevention tools. Supervisors now conduct the infection prevention exercise only if they judge that it is needed after having observed providers' performance during service delivery. Instead of conducting the entire COPE exercise, supervisors now reconvene with the providers and community representatives who developed the action plan during the last visit. Participants are asked to recall the discussion on rights-based service delivery and supervisors provide reorientation where necessary. Participants review the action plan to identify progress and determine further steps towards completion. The flexibility to choose different tools fosters comprehensive evaluation of reproductive health care. At the same time, it enables supervisors to focus on the areas of service delivery that are most in need of improvement.

## Conclusion

Formative supervision in Senegal can be sustained only with local leadership. There are more than 300 health facilities in the six intervention regions. As the sole implementing agency of formative supervision in Senegal, organizing visits to all the facilities was a significant challenge for MSH. Although the financial, technical, and human resources to lead supervision exists among many district health authorities, the leadership required to drive this process is often lacking. However, the example of several districts in the regions of Dakar and Ziguinchor, where some district health management teams have assumed full responsibility for the formative supervision process demonstrates that formative supervision *can *be locally sustained. District and regional health management personnel must be prepared not only to actively participate in the mobilization of resources but also to organize the supervision process and disseminate results in a timely manner. Informed and active local leaders are essential to mobilize the resources needed to continue formative supervision. The participation of community representatives and local health committees in the financial and logistical support of health facilities is also crucial.

Although challenges remain in the implementation and scaling up of this model, the data so far suggests that formative supervision offers immediate benefits to health providers and communities. For programmers engaged in quality improvement for reproductive health care, formative supervision offers an approach to obtaining crucial data that can be adapted according to local contexts and resources. MSH's experience in Senegal can serve as an example to advocate policymakers for resources to support formative supervision.

## Competing interests

The author(s) declare that they have no competing interests.

## Authors' contributions

SS, PM and ML participated in the analysis of supervision data and the revision of the article text. SS researched the literature, translated the examples of supervision tools and wrote the article text. All authors have read and approved the final manuscript.
